# Syphilitic Aortic Aneurysm: A Rare Entity in the Era of Antibiotics

**DOI:** 10.7759/cureus.13647

**Published:** 2021-03-01

**Authors:** Fizah Chaudhary, Armaghan Faghihimehr, Yogesh Subedi, Seyed Mohammad Hodanazari, Muhammad N Yousaf

**Affiliations:** 1 Medicine, MedStar Union Memorial Hospital, Baltimore, USA; 2 Medicine, MedStar Franklin Square Medical Center, Baltimore, USA; 3 Medicine, MedStar Harbor Hospital, Baltimore, USA; 4 Radiology, Virginia Commonwealth University Medical Center, Richmond, USA; 5 Medicine, MedStar Good Samaritan Hospital, Baltimore, USA

**Keywords:** syphilis, aortic aneurysm, aortitis, aortic aneurysm surgery

## Abstract

A thoracic aortic aneurysm is a rare entity of tertiary syphilis in the era of antibiotics. The diagnosis of the aortic aneurysm due to tertiary syphilis may be challenging due to deceptive clinical presentation and rarity of the disease in the western world. We report the case of a 59-year-old man, who presents with worsening shortness of breath and was found to have a large ascending aortic aneurysm on computed tomography angiogram (CTA) of the chest. Further workup demonstrated a positive syphilis test. Untreated earlier stages of syphilis attribute to the development of the ascending aortic aneurysm. The patient was medically treated with IV penicillin and underwent surgical repair of the aortic aneurysm. Histopathology confirmed the diagnosis of syphilitic aortitis. Tertiary syphilis often presents several years after initial infection and usually after a latent phase, making it difficult to diagnose. Syphilitic aortic aneurysms may result in a high mortality rate in untreated patients. Therefore, a high index of suspicion is required for the early recognition of a syphilitic aortic aneurysm. Early treatment with antibiotic therapy and surgical repair of syphilitic aortic aneurysms can prevent life-threatening complications.

## Introduction

The incidence of syphilis peaked during the second world war [1]. Widespread use of antibiotics for the management of earlier stages of syphilis and coincidental treatment of other infectious conditions with anti-treponemal antibiotic therapy has nearly eradicated the cases of tertiary syphilis from western countries [2]. Since the development of penicillin, syphilis cases of all stages dropped by 95% from 1943 to 2000 in the United States [3]. Cardiovascular syphilis generally manifests in the fourth to fifth decade of life, around 15-30 years after the initial infection and the majority of patients remain asymptomatic [4,5]. In untreated patients, *Treponema pallidum* inoculates into the aortic root several years after primary syphilis, causing an aortic root aneurysm. It is a serious complication that may result in high mortality without surgical repair [6]. We describe an atypical case of ascending aortic aneurysm due to tertiary syphilis in the United States which is extremely rare and may create diagnostic challenges for the physicians.

## Case presentation

**Figure 1:**
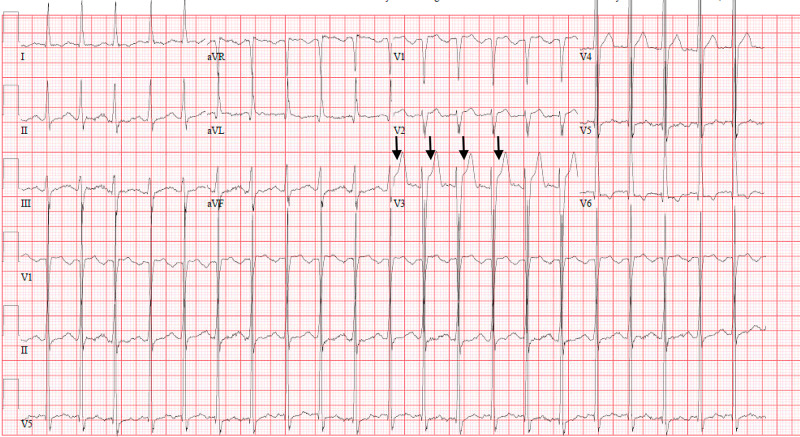
Electrocardiogram Anteroseptal ST elevation (arrows) without acute ischemic changes

**Figure 2:**
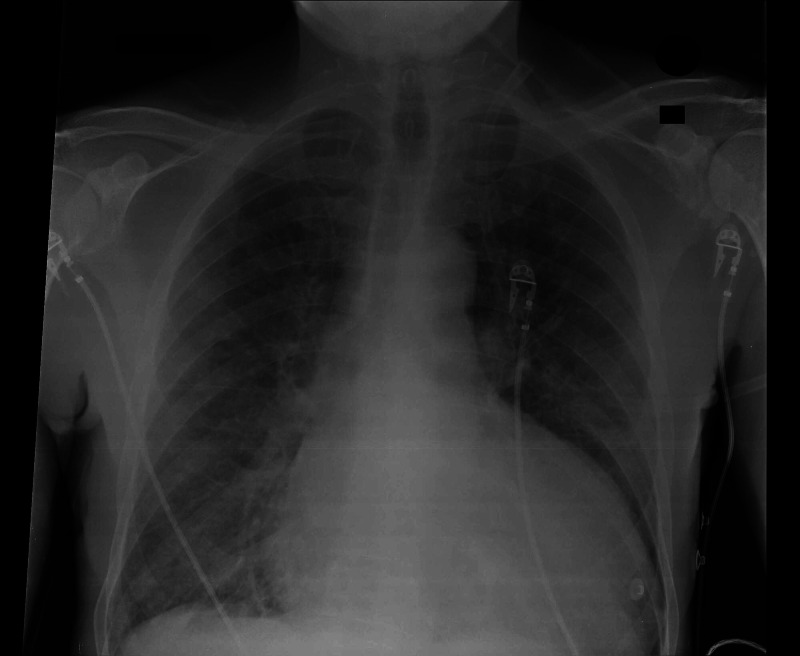
Chest radiograph The chest radiograph showing cardiomegaly and diffuse bilateral pulmonary infiltrates

**Figure 3:**
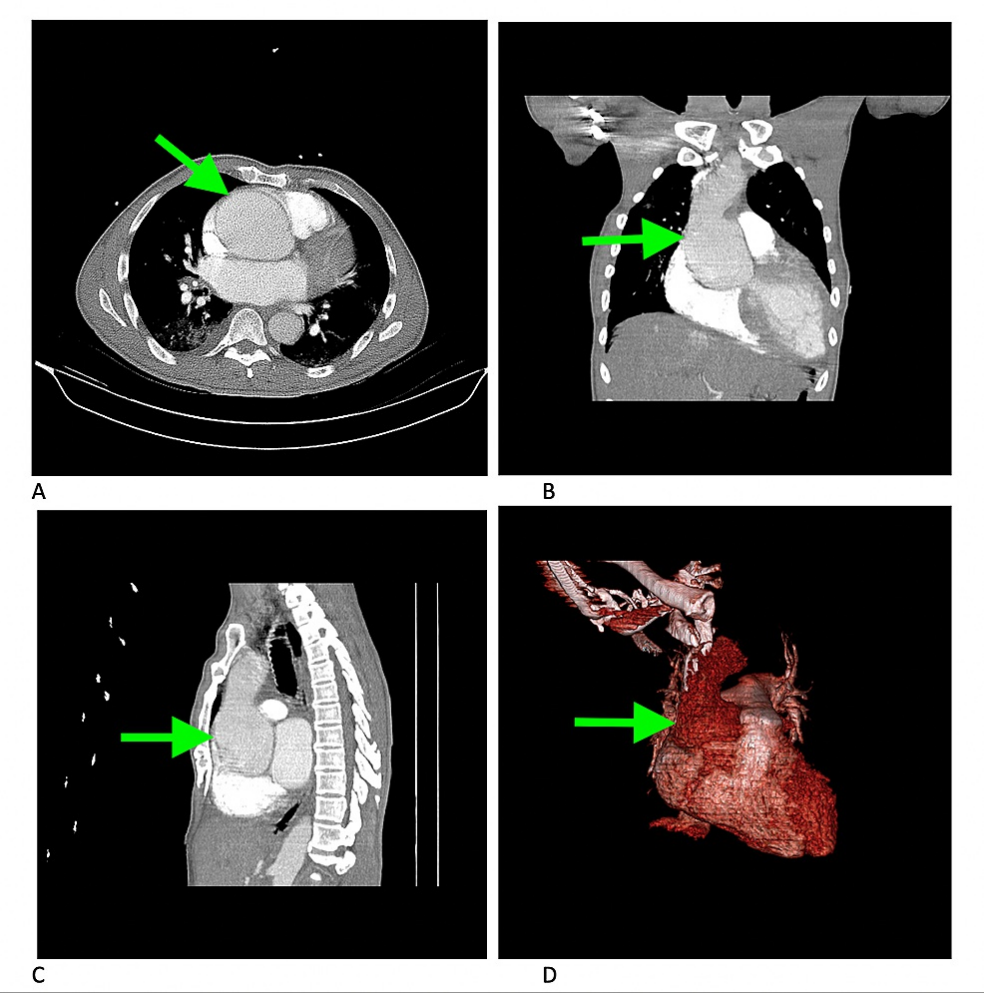
Chest CT angiography CT angiography of the chest demonstrating ascending aortic aneurysm (arrows) in axial (A), coronal (B), sagittal (C), and 3D reconstruction view (D)

A 59-year-old man presented to the emergency room with worsening shortness of breath and orthopnea for two weeks. He smokes five to six cigarettes daily for 10 years, denied alcohol drinking and recreational drug use. He is a retired accountant by profession, lives home independently with unknown history of sexually transmitted infections. During the presentation to the emergency room, he was hypotensive with a blood pressure of 96/51 mm Hg, heart rate 62/min, respiratory rate 20/min saturating 90% on room air. On examination, the patient was in acute respiratory distress, with an increased work of breathing, however, no wheezing or crackles on auscultation. Cardiac examination was remarkable for a 2/6 systolic ejection murmur and 2/6 diastolic murmur at the right upper sternal border. There was no jugular venous distension, peripheral edema, or abdominal tenderness. Laboratory workup revealed elevated N-terminal pro-B-type natriuretic peptide (NT-ProBNP) 8060 pg/mL (<177 pg/mL) troponin 0.141 ng/mL (0.000-0.045 ng/mL) peaked at 0.294 ng/mL. Blood chemistry and coagulation testing were unremarkable. Electrocardiogram (EKG) showed isolated ST elevation in lead V3, which was not correlating with acute ischemic changes (Figure 1). The chest radiograph revealed cardiomegaly and bilateral pulmonary infiltrates (Figure 2). Computed tomography angiogram (CTA) of the chest was unremarkable for pulmonary embolism but demonstrated a large aortic root aneurysm measuring 5.9 cm (Figure 3).

**Figure 4:**
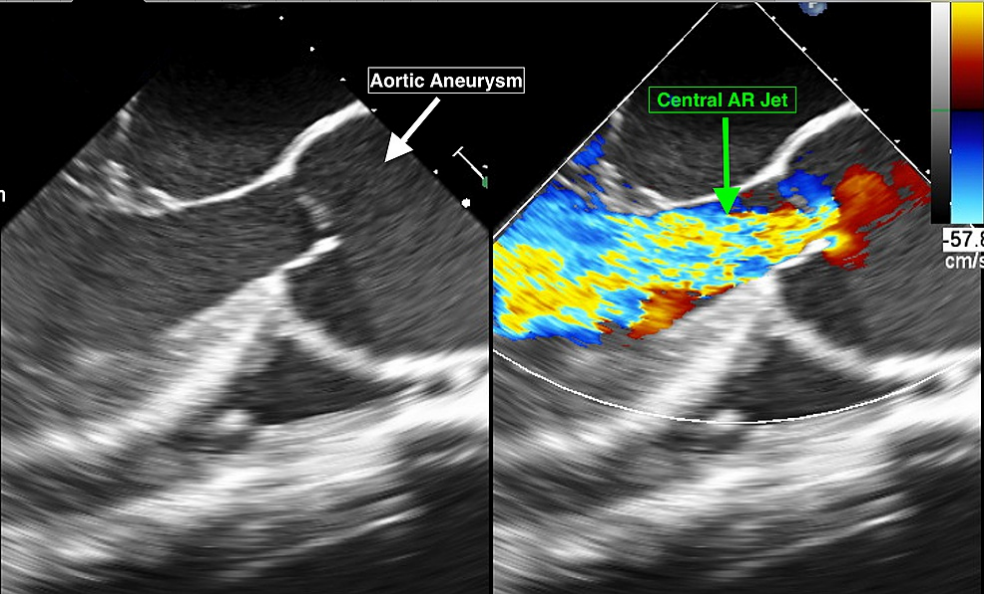
Transthoracic echocardiogram Transthoracic echocardiogram showing larger aortic root aneurysm (white arrow) with a central jet sign (green arrow) indicating severe aortic regurgitation (AR)

**Figure 5:**
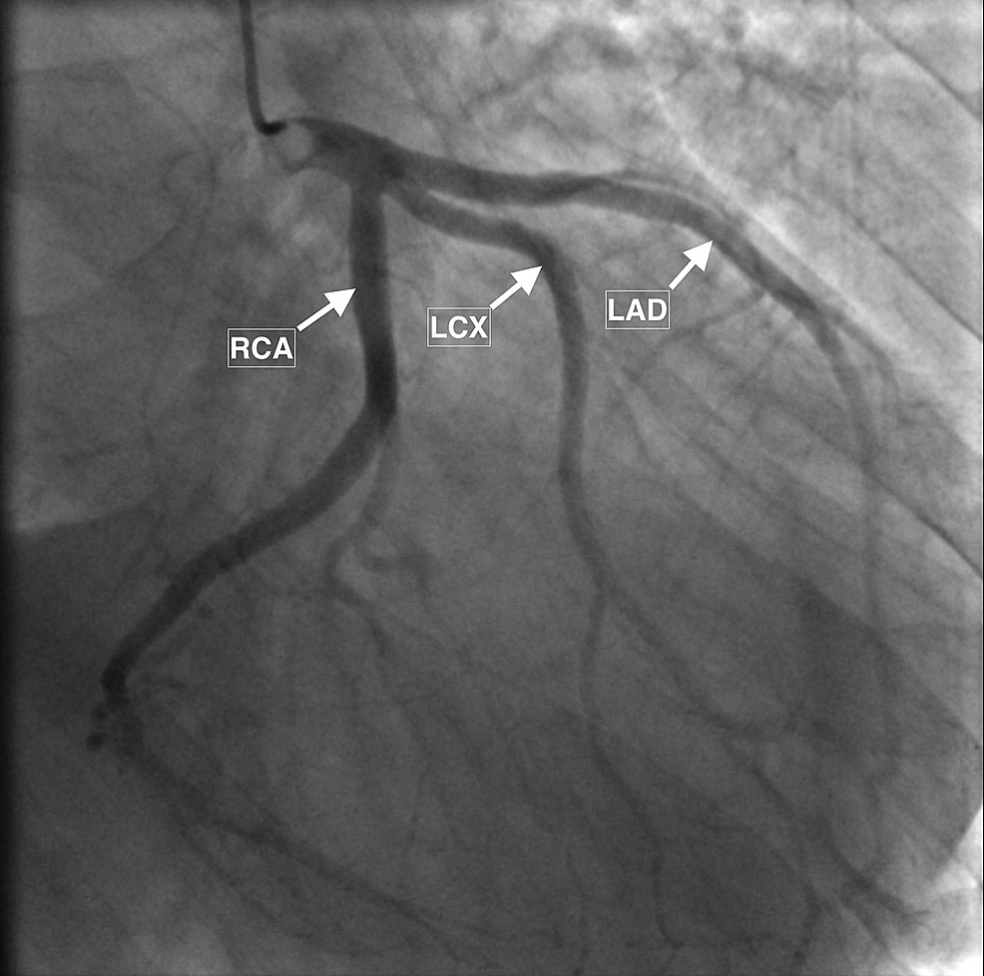
Coronary angiogram Coronary angiogram showing patent right coronary artery (RCA), left anterior descending (LAD), and left circumflex (LCX) coronary arteries without evidence of coronary flow limitations or obstruction

Transthoracic echocardiography revealed severe left ventricular dilation with reduced ejection fraction (30-35%) and severe aortic insufficiency as indicated with a large central jet sign (Figure 4). There was no evidence of coronary flow limitation or obstruction on the coronary angiogram (Figure 5).

**Figure 6:**
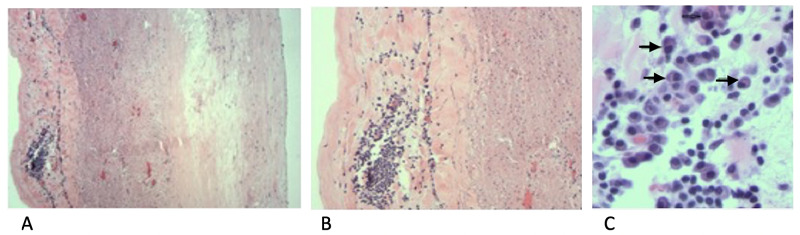
Histopathological examination of the aortic wall H&E stained pathological images with 40× (A), 100× (B), and 400× (C) magnification of the aortic root sample showing chronic inflammatory cells (plasma cells) in the adventitia marked by arrows

Workup for the etiology of aortic root aneurysms was only remarkable for a positive syphilis screening test that was confirmed with a positive *T. pallidum* particle agglutination assay (TP-PA). Testing for hepatitis B, hepatitis C, and HIV was negative. The patient was diagnosed with tertiary cardiovascular syphilis without evidence of neurosyphilis. The patient was treated with three doses of 2.4 million/unit intravenous (IV) penicillin-G and underwent surgical excision of aortic root aneurysm, aortic valve, aortic root replacement with 27 mm mosaic ultra bioprosthetic valve and 30 mm gelweave graft. Inspection of excised aortic root revealed aortic dissection within the aneurysm and marked inflammation along the left lateral wall of the aneurysm extending to the normal ascending aorta. Histopathological examination of the specimen demonstrated chronic inflammatory cells including lymphocytes, plasma cells in the fibrotic adventitia, and focal calcifications with myxoid degeneration of aortic root that confirmed the diagnosis of syphilitic aortitis (Figure 6).

## Discussion

Tertiary syphilis is a non-contagious stage of the disease that can often present several years after untreated infection with *T. pallidum* in early or latent stages. In the pre-antibiotic era, one-third of patients with untreated latent syphilis progress to tertiary syphilis [1]. A persistent inflammation of small and large vessels results in cardiovascular manifestations of tertiary syphilis. The predilection of spirochetes into the small vessels of the vasa vasorum leads to adventitial chronic inflammation, particularly involving the small arteries and arterioles that perfuse the media. This results in the ischemic injury of the aortic media with variable loss of smooth muscle cells and associated parenchyma. Destruction of aortic media, focal necrosis surrounded by palisading macrophages (also known as microgummas) and calcification develop within fibrotic tissues, creates fusiform or saccular aneurysm due to loss of elastic recoil of the aorta [7,8].

Cardiovascular syphilis classically involves thoracic ascending aorta (50%) because predominant lymphatic enrichment of this segment predisposes to mesoaortitis [5,6]. The aortic arch (35%) and descending segment of the aorta (15%) are less commonly involved [5,6]. Uncomplicated syphilitic aortitis is the most common (70-80%) manifestation of cardiovascular syphilis and 10% of these patients develop fusiform or saccular aortic aneurysm, aortic regurgitation, and stenosis of the coronary ostium [6,9]. Aneurysmal dilation may involve the aortic valve annulus causing chronic aortic valvular insufficiency leading to massive cardiac enlargement also known as “cor bovinum” (cow’s heart) [8]. The majority of patients are asymptomatic or identified incidentally on radiological imaging of the chest. Symptomatic patients usually present due to aneurysmal compression of surrounding structures such as dysphagia, dyspnea, hoarseness, cough, and superior vena cava compression syndrome. Dissection of syphilitic aortic aneurysm is extremely rare because of the fibrotic and calcified wall of the aneurysm, however, mortality due to fatal aneurysmal rupture has been reported [2,9]. Patients with untreated cardiovascular syphilis are at the risk of thrombosis and distal embolization that may result in a high rate of mortality due to acute coronary syndrome, cerebrovascular accidents, and visceral or limb ischemia [6,8,10].

The diagnosis of the aortic aneurysm due to tertiary syphilis may be challenging due to deceptive clinical presentation and rarity of the disease in the western world. Routine serologic testing of syphilis is not performed in such cases; however, a high index of suspicion is required for early recognition of a syphilitic aortic aneurysm in high-risk individuals such as homosexual, bisexual, or polygamous individuals with high-risk sexual behaviors [11]. The sensitivity of non-treponemal testing such as rapid plasma regain (RPR) and VDRL is 71-73% indicating approximately 25% of cases may be negative due to the low titer of circulating antibodies in late stages of syphilis [11-13]. In patients with aortic aneurysms, serologic confirmation of late stages of syphilis is required with treponemal specific tests such as TP-PA, microhemagglutination assay (MHA-TP), or fluorescent treponemal antibody absorption test (FTA-ABS). TP-PA is 87-100% sensitive, 100% specific, while MHA-TP and FTA-ABS are 97% and 85-93% sensitive, 99% and 87-100% specific, respectively [14]. In asymptomatic patients, the chest radiograph may identify an aortic aneurysm, however, a CTA chest is required to determine the size, anatomical involvement of the aorta, and planning for surgical management of aneurysm. Preoperative coronary angiogram and echocardiography are required to identify coexisting aortic insufficiency and limitation of coronary flow [2,9]. Late latent stages of syphilis of unknown duration are treated with three doses of intramuscular benzathine penicillin G (2.4 million units) at one-week intervals. Surgical repair such as resection of dilated segment of aortic aneurysm and placement of the synthetic vascular graft is the definitive management of this entity [9]. In surgically untreated patients with a thoracic aortic aneurysm, five-year survival is only 20% that improves to 60-70% after elective surgical repair [15].

In the past decade, several cases of syphilitic thoracic aortic aneurysm have been reported with variable clinical presentation such as simultaneous involvement of the brain, spinal cord (neurosyphilis), lungs (pulmonary syphilis), and rupture or dissection of thoracic aortic aneurysm (cardiovascular syphilis) [5,7,16-20]. Our case is a classic presentation of isolated cardiovascular syphilis presented with a large thoracic aortic aneurysm confirmed with TP-PA testing and radiological imaging. Medical management with penicillin-G and surgical resection and replacement of aortic root resulted in complete resolution of the patient’s symptoms.

## Conclusions

The diagnosis of cardiovascular syphilis may be challenging due to atypical presentation. High-risk individuals with a thoracic aortic aneurysm should be tested for syphilis screening followed by the treponemal specific test as early diagnosis and treatment of tertiary syphilis with antibiotic therapy and subsequent surgical repair of syphilitic aortic aneurysms can prevent fatal complications and mortality.
